# The post-Higgs MSSM scenario: habemus MSSM?

**DOI:** 10.1140/epjc/s10052-013-2650-0

**Published:** 2013-11-27

**Authors:** A. Djouadi, L. Maiani, G. Moreau, A. Polosa, J. Quevillon, V. Riquer

**Affiliations:** 1 Laboratoire de Physique Théorique, Université Paris–Sud and CNRS, 91405 Orsay, France; 2 Department of Physics and INFN, “Sapienza” Universtà di Roma, Pizzale Aldo Moro 5, 00185 Roma, Italy; 3 Theory Unit, CERN, 1211 Genève 23, Switzerland

## Abstract

We analyze the Minimal Supersymmetric extension of the Standard Model that we have after the discovery of the Higgs boson at the LHC, the hMSSM (habemus MSSM?), i.e. a model in which the lighter *h* boson has a mass of approximately 125 GeV which, together with the non-observation of superparticles at the LHC, indicates that the SUSY-breaking scale *M*_*S*_ is rather high, *M*_*S*_≳1 TeV. We first demonstrate that the value *M*_*h*_≈125 GeV fixes the dominant radiative corrections that enter the MSSM Higgs boson masses, leading to a Higgs sector that can be described, to a good approximation, by only two free parameters. In a second step, we consider the direct supersymmetric radiative corrections and show that, to a good approximation, the phenomenology of the lighter Higgs state can be described by its mass and three couplings: those to massive gauge bosons and to top and bottom quarks. We perform a fit of these couplings using the latest LHC data on the production and decay rates of the light *h* boson and combine it with the limits from the negative search of the heavier *H*,*A* and *H* ^±^ states, taking into account the current uncertainties.

## Introduction

The observation at the LHC of a Higgs particle with a mass of 125 GeV [1, 2] has important implications for Supersymmetric (SUSY) and, in particular, for the Minimal Supersymmetric Standard Model (MSSM). In this extension, the Higgs sector consists of two scalar doublet fields *H*_*u*_ and *H*_*d*_ that lead, after electroweak symmetry breaking, to five Higgs states, two CP-even *h* and *H*, a CP-odd *A* and two charged *H* ^±^ bosons [3–6]. At tree level, the masses of these particles and their mixings are described by only two parameters usually chosen to be the ratio of the vacuum expectations values of the two doublet fields tan*β*=*v*_*d*_/*v*_*u*_ and the mass *M*_*A*_ of the pseudoscalar Higgs boson. However, as is well known, the radiative corrections play a very important role as their dominant component grows like the fourth power of the top quark mass, logarithmically with the supersymmetry breaking scale *M*_*S*_ and quadratically with the stop mixing parameter *A*_*t*_; see e.g. Refs. [6–11].

The impact of the Higgs discovery is two-fold. On the one hand, it gives support to the MSSM in which the lightest Higgs boson is predicted to have a mass below ≈130 GeV when the radiative corrections are included [6–11]. On the other hand, the fact that the measured value *M*_*h*_≈125 GeV is close to this upper mass limit implies that the SUSY-breaking scale *M*_*S*_ might be rather high. This is backed up by the presently strong limits on supersymmetric particle masses from direct searches that indicate that the SUSY partners of the strongly interacting particles, the squarks and gluinos, are heavier than ≈1 TeV [12]. Hence, the MSSM that we currently have, and that we call hMSSM (habemus MSSM?) in the subsequent discussion, appears to have *M*_*h*_≈125 GeV and *M*_*S*_≳1 TeV.

It was pointed out in Refs. [13–15] that when the information *M*_*h*_=125 GeV is taken into account, the MSSM Higgs sector with solely the dominant radiative correction to the Higgs boson masses included, can be again described with only the two free parameters tan*β* and *M*_*A*_ as it was the case at tree level. In other words, the dominant radiative corrections that involve the SUSY parameters are fixed by the value of *M*_*h*_. In this paper, we show that to a good approximation, this remains true even when the full set of radiative corrections to the Higgs masses at the two-loop level is included. This is demonstrated in particular by performing a full scan on the MSSM parameters that have an impact on the Higgs sector such as for instance tan*β* and the stop and sbottom mass and mixing parameters. The subleading radiative corrections are shown to have little impact on the mass and mixing of the heavier Higgs bosons when these SUSY parameters are varied in a reasonable range.

Nevertheless, there are also possibly large direct SUSY radiative corrections that modify the Higgs boson couplings and which might alter this simple picture. Among such corrections are, for instance, the stop contribution [16–19] to the dominant Higgs production mechanism at the LHC, the gluon fusion process *gg*→*h*, and to the important decay into two photons *h*→*γγ*, and the additional one-loop vertex corrections to the *h* couplings to *b*-quarks that grow with tan*β* [20]. In the most general case, besides *M*_*h*_, seven couplings need to be considered to fully describe the properties of the observed *h* boson: those to gluons, photons, massive gauge bosons, *t*,*b*,*c*-quarks and *τ* leptons. However, we show that given the accuracy that is foreseen at the LHC, a good approximation is to consider the three effective couplings to *t*,*b* quarks and to *V*=*W*/*Z* bosons, *c*_*t*_,*c*_*b*_ and *c*_*V*_, as it was suggested in Ref. [21]. Following the approach of Refs. [22–24] for the inclusion of the current theoretical and experimental uncertainties, we perform a fit of these three couplings using the latest LHC data on the production and decay rates of the lighter *h* boson and the limits from the negative search of the heavier *H*,*A* and *H* ^±^ MSSM states.

Almost one year after the Higgs discovery at the LHC, these two aspects will be discussed in the next two sections. A brief discussion and a conclusion are given in Sect. 4 and a short Appendix collects a set of formulas used in this analysis.

## Post Higgs discovery parametrization of radiative corrections

In the CP-conserving MSSM,^1^ the tree-level CP-even *h* and *H* masses depend on *M*_*A*_, tan*β* and the *Z* boson mass. However, many parameters of the MSSM such as the SUSY scale, taken to be the geometric average of the stop masses $M_{S}= \sqrt{m_{\tilde{t}_{1}} m_{\tilde{t}_{2}} }$, the stop/sbottom trilinear couplings *A*_*t*/*b*_ or the higgsino mass *μ* enter *M*_*h*_ and *M*_*H*_ through radiative corrections. In the basis (*H*_*d*_,*H*_*u*_), the CP-even Higgs mass matrix can be written as

1 $$\begin{aligned} M_{S}^2 =& M_{Z}^2 \left ( \begin{array}{c@{\quad }c} c^2_\beta& -s_\beta c_\beta\\ -s_\beta c_\beta& s^2_\beta\\ \end{array} \right ) +M_{A}^2 \left ( \begin{array}{c@{\quad }c} s^2_\beta& -s_\beta c_\beta\\ -s_\beta c_\beta& c^2_\beta\\ \end{array} \right ) \\ &{} + \left ( \begin{array}{c@{\quad }c} \varDelta \mathcal{ M}_{11}^2 & \varDelta \mathcal{ M}_{12}^2 \\ \varDelta \mathcal{ M}_{12}^2 &\varDelta \mathcal{ M}_{22}^2 \\ \end{array} \right ) \end{aligned}$$

where we use the short-hand notation *s*_*β*_≡sin*β* etc. and have introduced the radiative corrections by a 2×2 general matrix $\varDelta \mathcal{ M}_{ij}^{2}$. One can then easily derive the neutral CP-even Higgs boson masses and the mixing angle *α* that diagonalizes the *h*,*H* states,^2^ $H= \cos\alpha H_{d}^{0} + \sin\alpha H_{u}^{0}$ and $h= -\sin\alpha H_{d}^{0} + \cos\alpha H_{u}^{0}$:

2 $$\begin{aligned} & M_{h/H}^2=\frac{1}{2} \Bigl( M_{A}^2+M_{Z}^2+ \varDelta \mathcal{ M}_{11}^2+ \varDelta \mathcal{ M}_{22}^2 \mp \sqrt{ M_{A}^4+M_{Z}^4-2 M_{A}^2 M_{Z}^2 c_{4\beta} +C} \Bigr) \end{aligned}$$

3 $$\begin{aligned} & \tan\alpha= \frac{2\varDelta \mathcal{ M}_{12}^2 - (M_{A}^2 + M_{Z}^2) s_{\beta}}{ \varDelta \mathcal{ M}_{11}^2 - \varDelta \mathcal{ M}_{22}^2 + (M_{Z}^2-M_{A}^2) c_{2\beta} + \sqrt{M_{A}^4 + M_{Z}^4 - 2 M_{A}^2 M_{Z}^2 c_{4\beta} + C}} \end{aligned}$$

$$\begin{aligned} C= 4 \varDelta \mathcal{ M}_{12}^4 + \bigl( \varDelta \mathcal{ M}_{11}^2 - \varDelta \mathcal{ M}_{22}^2 \bigr)^2 - 2 \bigl(M_{A}^2 - M_{Z}^2\bigr) \bigl( \varDelta \mathcal{ M}_{11}^2 - \varDelta M_{22}^2\bigr) c_{2\beta} - 4 \bigl(M_{A}^2 + M_{Z}^2\bigr) \varDelta \mathcal{ M}_{12}^2 s_{2\beta} \end{aligned}$$

In previous analyses [13–15], we have assumed that in the 2×2 matrix for the radiative corrections, only the $\varDelta \mathcal{ M}^{2}_{22}$ entry which involves the by far dominant stop–top sector correction, is relevant, $\varDelta \mathcal{ M}^{2}_{22} \gg\varDelta \mathcal{ M}^{2}_{11}, \varDelta \mathcal{ M}^{2}_{12}$. This occurs, for instance, in the so-called *ϵ* approximation [7–9] and its refinements [10, 11] that are given in Eqs. (A.2) and (A.3) of the Appendix. In this case, one can simply trade $\varDelta \mathcal{ M}^{2}_{22}$ for the by now known *M*_*h*_ using

4 $$\begin{aligned} \varDelta \mathcal{ M}^{2}_{22}= \frac{M_{h}^2(M_{A}^2 + M_{Z}^2 -M_{h}^2) - M_{A}^2 M_{Z}^2 c^{2}_{2\beta} }{ M_{Z}^2 c^{2}_{\beta} +M_{A}^2 s^{2}_{\beta} -M_{h}^2} \end{aligned}$$

In this case, one can simply write *M*_*H*_ and *α* in terms of *M*_*A*_,tan*β* and *M*_*h*_:

5 $$\begin{aligned} & \mathrm{hMSSM}{:} \\ &\quad M_{H}^2 = \frac{(M_{A}^2+M_{Z}^2-M_{h}^2)(M_{Z}^2 c^{2}_{\beta}+M_{A}^2 s^{2}_{\beta}) - M_{A}^2 M_{Z}^2 c^{2}_{2\beta} }{M_{Z}^2 c^{2}_{\beta}+M_{A}^2 s^{2}_{\beta} - M_{h}^2} \\ & \quad \alpha= -\arctan \biggl(\frac{ (M_{Z}^2+M_{A}^2) c_{\beta} s_{\beta}}{M_{Z}^2 c^{2}_{\beta}+M_{A}^2 s^{2}_{\beta} - M_{h}^2} \biggr) \end{aligned}$$

In this section, we will check the validity of the $\varDelta \mathcal{ M}^{2}_{11}= \varDelta \mathcal{ M}^{2}_{12}=0$ approximation. To do so, we first consider the radiative corrections when the subleading contributions proportional to *μ*,*A*_*t*_ or *A*_*b*_ are included in the form of Eq. (A.4) of the Appendix, which is expected to be a good approximation [6, 25], and in which one has $\varDelta \mathcal{ M}^{2}_{11} \neq{\varDelta \mathcal{ M}^{2}_{12} \neq0}$.

**Fig. 1:**
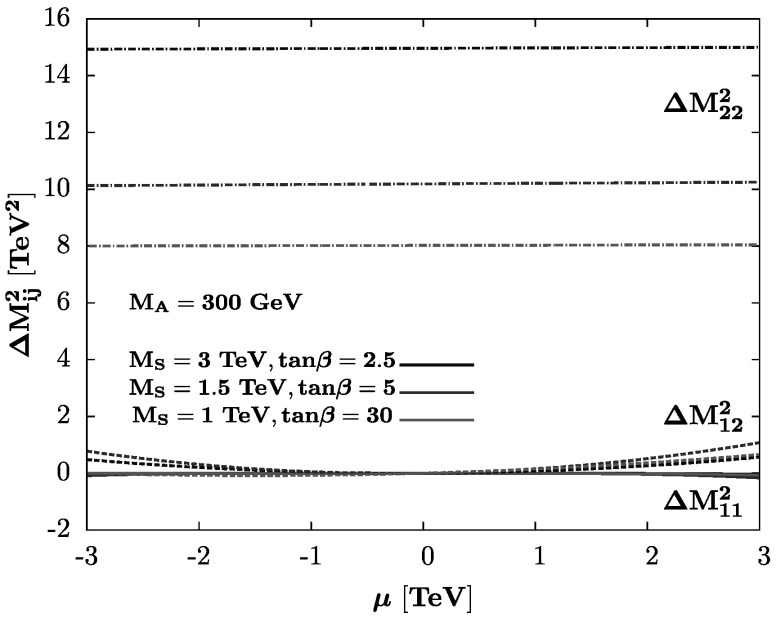
The entries $\varDelta \mathcal{ M}^{2}_{11}$ (*solid*), $\varDelta \mathcal{ M}^{2}_{12}$ (*dashed*), and $\varDelta \mathcal{ M}^{2}_{22}$ (*dotted–dashed lines*) of the radiative corrections matrix as functions of *μ* with a fixed *M*_*A*_=300 GeV for three different (*M*_*S*_,tan*β*) sets and *A*_*t*_ such that it accommodates the mass range *M*_*h*_=123–129 GeV

As a first step we only consider the stop-top sector corrections which enter the $\varDelta \mathcal{ M}^{2}_{ij}$ terms and confront in Fig. 1, the values of $\varDelta \mathcal{ M}^{2}_{11}$, $\varDelta \mathcal{ M}^{2}_{12}$ to $\varDelta \mathcal{ M}^{2}_{22}$ for three different scenarios with *M*_*A*_=300 GeV (i.e. before the onset of the decoupling regime *M*_*A*_≫*M*_*Z*_): *M*_*S*_=3 TeV and tan*β*=2.5, *M*_*S*_=1.5 TeV and tan*β*=5, *M*_*S*_=1 TeV and tan*β*=30. The parameter *A*_*t*_ is adjusted in order to accommodate a light Higgs boson with a mass *M*_*h*_=126±3 GeV, including an expected theoretical and experimental uncertainty of 3 GeV [26–28]. One observes that for reasonable *μ* values, one obtains naturally $\varDelta \mathcal{ M}^{2}_{11},\varDelta \mathcal{ M}^{2}_{12} \ll\varDelta \mathcal{ M}^{2}_{22}$.

We have verified that the situation is not very different if the corrections in the sbottom sector are also included: assuming *A*_*b*_=*A*_*t*_, we also obtain the hierarchy $\varDelta \mathcal{ M}^{2}_{11},\varDelta \mathcal{ M}^{2}_{12} \ll\varDelta \mathcal{ M}^{2}_{22}$ for *μ*≲3 TeV even for tan*β*=30 where contributions ∝*μ*tan*β* become important.

**Fig. 2:**
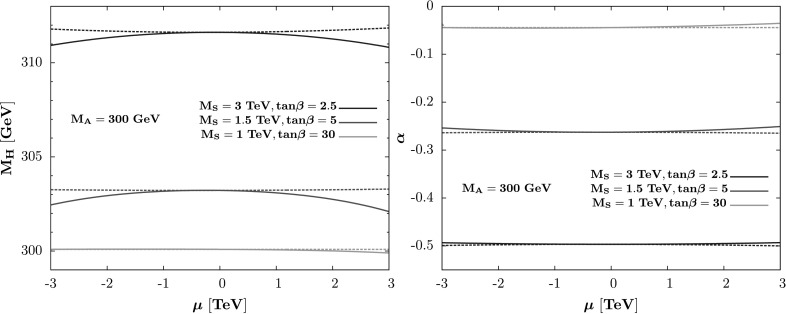
The mass of the heavier CP-even *H* boson (*left*) and the mixing angle *α* (*right*) as a function of *μ* with (*solid lines*) and without (*dashed*) the off-diagonal components for *M*_*A*_=300 GeV and three (*M*_*S*_,tan*β*) sets. *A*_*t*_ is such that *M*_*h*_=123–129 GeV and *A*_*b*_=0

Taking into account only the dominant top–stop radiative corrections in the approximations of Eq. (A.4), Fig. 2 displays the mass of the heavy CP-even Higgs state (left) and the mixing angle *α* (right) as a function of *μ* when $\varDelta \mathcal{ M}^{2}_{11}$ and $\varDelta \mathcal{ M}^{2}_{12}$ are set to zero (dashed lines) and when they are included (solid lines). We have assumed the same (*M*_*S*_,tan*β*) sets as above and for each value of *μ*, we calculate “approximate” and “exact” *M*_*H*_ and *α* values assuming *M*_*h*_=126±3 GeV. Even for large values of the parameter *μ* (but *μ*≲3 TeV), the relative variation for *M*_*H*_ never exceeds the 0.5 % level while the variation of the angle *α* is bounded by *Δα*≲0.015. Hence, in this scenario for the radiative corrections, the approximation of determining the parameters *M*_*H*_ and *α* from tan*β*,*M*_*A*_ and the value of *M*_*h*_ is extremely good. We have again verified that it stays the case when the corrections in the sbottom sector, with *A*_*b*_=*A*_*t*_, are included.

We should note that for higher *M*_*A*_ values, *M*_*A*_≳300 GeV, the approximation is even better as we are closer to the decoupling limit in which one has *M*_*H*_=*M*_*A*_ and $\alpha= \frac{\pi}{2}-\beta$. Lower values, *M*_*A*_≲300 GeV, are disfavored by the observed *h* rates [14, 15] as seen later.

In order to check more thoroughly the impact of the subleading corrections $\varDelta \mathcal{ M}^{2}_{11}$, $\varDelta \mathcal{ M}^{2}_{12}$, we perform a scan of the MSSM parameter space using the program SuSpect [29, 30] in which the full two-loop radiative corrections to the Higgs sector are implemented. For a chosen (tan*β*, *M*_*A*_) input set, the soft-SUSY parameters that play an important role in the Higgs sector are varied in the following ranges: |*μ*|≤3 TeV, |*A*_*t*_,*A*_*b*_|≤3*M*_*S*_, 1 TeV≤*M*_3_≤3 TeV and 0.5 TeV≤*M*_*S*_≤3 TeV (≈3 TeV is the scale up to which programs such as SuSpect are expected to be reliable). We assume the usual relation between the weak scale gaugino masses 6*M*_1_=3*M*_2_=*M*_3_ and set *A*_*u*_,*A*_*d*_,*A*_*τ*_=0 (these last parameters have little impact).

We have computed the MSSM Higgs sector parameters all across the parameter space selecting the points which satisfy the constraint 123≤*M*_*h*_≤129 GeV. For each of the points, we have compared the Higgs parameters to those obtained in the simplified MSSM approximation, $\varDelta \mathcal{ M}^{2}_{11} = \varDelta \mathcal{ M}^{2}_{12} = 0$, with the lightest Higgs boson mass as input. We also required *M*_*h*_ to lie in the range 123–129 GeV, but allowed it to be different from the one obtained in the “exact” case $\varDelta \mathcal{ M}^{2}_{11}, \varDelta \mathcal{ M}^{2}_{12} \neq0$.

**Fig. 3:**
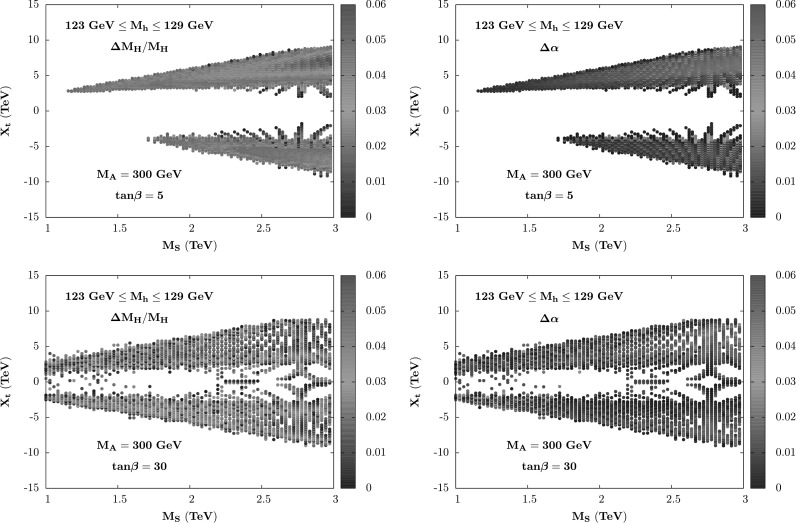
The variation of the mass *M*_*H*_ (*left*) and the mixing angle *α* (*right*), are shown as separate *vertical colored scales*, in the plane [*M*_*S*_,*X*_*t*_] when the full two loop corrections are included with and without the subleading matrix elements $\varDelta \mathcal{ M}^{2}_{11}$ and $\varDelta \mathcal{ M}^{2}_{12}$. We take *M*_*A*_=300 GeV, tan*β*=5 (*top*) and 30 (*bottom*) and the other parameters are varied as stated in the text

For the mass *M*_*H*_ and the angle *α*, we display in Fig. 3 the difference between the values obtained when the two possibilities $\varDelta \mathcal{ M}^{2}_{11} = \varDelta \mathcal{ M}^{2}_{12} = 0$ and $\varDelta \mathcal{ M}^{2}_{11}, \varDelta \mathcal{ M}^{2}_{12} \neq 0$ are considered. This is shown in the plane [*M*_*S*_,*X*_*t*_] with *X*_*t*_=*A*_*t*_−*μ*cot*β* when all other parameters are scanned as above. Again, we have fixed the pseudoscalar Higgs mass to *M*_*A*_=300 GeV and used the two representative values tan*β*=5 and 30. We have adopted the conservative approach of plotting only points which maximize these differences.

In all cases, the difference between the two *M*_*H*_ values is very small (in fact, much smaller than the total decay width *Γ*_*H*_), less than a few percent, while for *α* the difference does not exceed ≈0.025 for low values of tan*β* but at high tan*β* values, one can reach the level of ≈0.05 in some rare situations (large values of *μ*, which enhance the *μ*tan*β* contributions). Nevertheless, at high enough tan*β*, we are far in the decoupling regime already for *M*_*A*_≳200 GeV and such a difference does not significantly affect the couplings of the *h* and *H* bosons which, phenomenologically, are the main ingredients.

Hence, even when including the full set of radiative corrections up to two loops, it is a good approximation to use Eqs. (5) to derive the parameters *M*_*H*_ and *α* in terms of the inputs tan*β*,*M*_*A*_ and the measured value of *M*_*h*_. In the case of the charged Higgs boson mass, the radiative corrections are much smaller for large enough *M*_*A*_ and one has, at the few percent level (which is again smaller than the total *H* ^±^ decay width), $M_{H^{\pm}} \simeq\sqrt{ M_{A}^{2} + M_{W}^{2}}$ except in very rare situations^3^ [31].

## Determination of the *h* boson couplings in a generic MSSM

A second important issue is the MSSM Higgs couplings. In principle and as discussed earlier, knowing two parameters such as the pair of inputs [tan*β*,*M*_*A*_] and fixing the value of *M*_*h*_ to its measured value, the couplings of the Higgs bosons, in particular *h*, to fermions and gauge bosons can be derived, including the generally dominant radiative corrections that enter in the MSSM Higgs masses. Indeed, in terms of the angles *β* and *α*, one has for the reduced couplings (i.e. normalized to their SM values) of the lighter *h* state to third generation *t*,*b* fermions and gauge bosons *V*=*W*/*Z*,

6 $$\begin{aligned} c_V^0 = \sin(\beta- \alpha) ,\qquad c_t^0 = \frac{\cos \alpha}{\sin\beta} , \qquad c_b^0 = - \frac{\sin \alpha}{\cos\beta} \end{aligned}$$

However, outside the regime in which the pseudoscalar *A* boson and some supersymmetric particles are very heavy, there are also direct radiative corrections to the Higgs couplings not contained in the mass matrix of Eq. (1). These can alter this simple picture.

First, in the case of *b*-quarks, additional one-loop vertex corrections modify the tree-level $h b \bar{b}$ coupling: they grow as *m*_*b*_ *μ*tan*β* and are thus very large at high tan*β*. The dominant component comes from the SUSY-QCD corrections with sbottom–gluino loops that can be approximated by $\varDelta _{b} \simeq 2\alpha_{s}/(3\pi) \times\mu m_{\tilde{g}} \tan\beta /{\rm max} (m_{\tilde{g}}^{2}, m_{\tilde{b}_{1}}^{2},m_{\tilde{b}_{2}}^{2}) $ [20].

Outside the decoupling regime, the $hb\bar{b}$ coupling receives the possibly large correction

7 $$\begin{aligned} & c_b \approx c_b^0 \times\bigl[1- \varDelta _b/(1+\varDelta _b) \times(1+ \cot \alpha\cot\beta) \bigr] \\ &\quad \mathrm{with}\ \tan\alpha\stackrel{M_A \gg M_Z}{\to}-1/\tan\beta \end{aligned}$$

which would significantly alter the partial width of the decay $h \to b\bar{b}$ that is, in principle, by far the dominant one and, hence, affect the branching fractions of all other decay modes.

In addition, the $ht\bar{t}$ coupling is derived indirectly from the *gg*→*h* production cross section and the *h*→*γγ* decay branching ratio, two processes that are generated via triangular loops. In the MSSM, these loops involve not only the top quark (and the *W* boson in the decay *h*→*γγ*) but also contributions from supersymmetric particles, if they are not too heavy. In the case of the *gg*→*h* process, only the contributions of stops is generally important. Including the latter and working in the limit $M_{h} \ll m_{t}, m_{\tilde{ t}_{1} }, m_{\tilde{ t}_{2} }$, the *hgg* amplitude can be (very well) approximated by the expression [16–18]

8 $$\begin{aligned} c_t \approx & c_t^0 \times \biggl[ 1 + \frac{m_t^2}{ 4 m_{\tilde{t}_1}^2 m_{\tilde{t}_2}^2 } \bigl( m_{\tilde{t}_1}^2 + m_{\tilde{t}_2}^2 \\ &{} - (A_t -\mu\cot\alpha) ( A_t+\mu\tan\alpha) \bigr) \biggr] \end{aligned}$$

which shows that indeed, $\tilde{t}$ contributions can be very large for sufficiently light stops and in the presence of large stop mixing. In the *h*→*γγ* decay rate, because the $t, \tilde{t}$ electric charges are the same, the $ht\bar{t}$ coupling is shifted by the same amount as above [19].

If one ignores the usually small $\tilde{b}$ contributions in the *gg*→*h* production and *h*→*γγ* decay processes (in the latter case, it is suppressed by powers of the *b* electric charge $e_{b}^{2}/e_{t}^{2} = \frac{1}{4}$ in addition) as well as the contributions of other SUSY particles such as charginos and staus in the *h*→*γγ* decay rate,^4^ the leading corrections to the $ht\bar{t}$ vertex can be simply accounted for by using the effective coupling given in Eq. (8); see e.g. Ref. [14].

Note that in the case of associated production of the *h* boson with top quarks, $gg/q\bar{q} \to h t\bar{t}$, it is the parameter $c_{t}^{0}$ which should be considered for the direct $ht\bar{t}$ coupling. However, for the time being (and presumably for a long time), the constraints on the *h* properties from this process are very weak as the cross section has very large uncertainties.

One also should note that the couplings of the *h* boson to *τ* leptons and charm quarks do not receive the direct corrections of, respectively, Eqs. (7) and (8) and one should still have $c_{c}=c_{t}^{0}$ and $c_{\tau}= c_{b}^{0}$. However, using *c*_*t*,*b*_ or $c_{t,b}^{0}$ in this case has almost no impact in practice as these couplings appear only in the branching ratios for the decays $h \to c\bar{c}$ and *τ* ^+^ *τ* ^−^ which are small, below 5 %, and the direct corrections cannot be very large (these are radiative corrections after all). One can thus, in a first approximation, ignore them and assume that *c*_*c*_=*c*_*t*_ and *c*_*τ*_=*c*_*b*_. Note that BR($h\to c\bar{c}$) cannot be measured at the LHC while the *h*→*τ* ^+^ *τ* ^−^ rate is presently measured only at the level of 40 % or so [34].

Another caveat is that possible invisible decays (which at present are probed directly only for rates that are at the 50 % to 100 % level [35]), can also affect the properties of the observed *h* particle. However, a large invisible rate implies that the neutralinos that are considered as the lightest SUSY particles, are relatively light and couple significantly to the *h* boson, a situation that is rather unlikely (if the LSP is very light, $2m_{\chi_{1}^{0}} \lesssim M_{h}$, it should be mostly bino-like and, hence, has very suppressed couplings to the Higgs bosons that prefer to couple to mixtures of higgsinos and gauginos; see for instance Ref. [19]).

In the case of large direct corrections, the Higgs couplings cannot be described only by the parameters *β* and *α* as in Eq. (6). One should consider at least three independent *h* couplings, namely *c*_*c*_=*c*_*t*_, *c*_*τ*_=*c*_*b*_ and $c_{V}=c_{V}^{0}$ as advocated in Ref. [21]. This is equivalent to excluding the *h*→*ττ* data from the global fit which, in practice, has no significant impact as the experimental error on the signal strength in this channel is presently large. Note that a future determination of the theoretically clean ratio of the $b\bar{b}$ and *τ* ^+^ *τ* ^−^ signals in *pp*→*hV* gives a direct access to the *Δ*_*b*_ correction outside the decoupling regime [22–24].

To study the *h* state at the LHC, we thus define the following effective Lagrangian:

9 $$\begin{aligned} \mathcal{ L}_h = & c_V g_{hWW} h W_{\mu}^+ W^{- \mu} + c_V g_{hZZ} h Z_{\mu}^0 Z^{0 \mu} \\ &{}- c_t y_t h \bar{t}_L t_R - c_t y_c h \bar{c}_L c_R - c_b y_b h \bar{b}_L b_R \\ &{} - c_b y_\tau h \bar{\tau}_L \tau_R + \mathrm{h.c.} \end{aligned}$$

where *y*_*t*,*c*,*b*,*τ*_=*m*_*t*,*c*,*b*,*τ*_/*v* are the SM Yukawa coupling constants in the mass eigenbasis (*L*/*R* indicates the fermion chirality and we consider only the heavy fermions that have substantial couplings to the Higgs boson), $g_{hWW} = 2M^{2}_{W}/v$ and $g_{hZZ} = M^{2}_{Z}/v$ are the electroweak gauge boson couplings and *v* is the Higgs vacuum expectation value.

We present the results for the fits of the Higgs signal strengths in the various channels

10 $$\begin{aligned} \mu_X \simeq& \sigma( pp \to h) \times{\rm BR}(h \to XX)/ \sigma( pp \to h)_{\rm SM} \\ &{} \times{\rm BR}(h \to XX)_{\rm SM} \end{aligned}$$

closely following the procedure of Refs. [22–24] but in the case of the phenomenological MSSM. All the Higgs production/decay channels are considered and the data used are the latest ones [34] using the full ≈25 fb^−1^ statistics for the *γγ*,*ZZ*,*WW* channels as well as the $h\to b\bar{b}$ and *ττ* modes for CMS, but only ≈17 fb^−1^ data for the ATLAS fermionic channels.

**Fig. 4:**
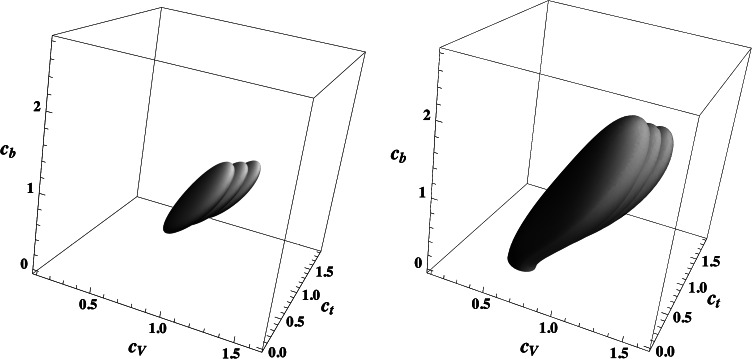
Best-fit regions at $68\ \%\ {\rm CL}$ (*green*, *left*) and $99\ \%\ {\rm CL}$ (*light gray*, *right*) for the Higgs signal strengths in the three-dimensional space [*c*_*t*_,*c*_*b*_,*c*_*V*_]. The three overlapped regions are associated to central and two extreme choices of the theoretical prediction for the Higgs rates

We have performed the appropriate three-parameter fit in the three-dimensional space^5^ [*c*_*t*_,*c*_*b*_,*c*_*V*_], assuming *c*_*c*_=*c*_*t*_ and *c*_*τ*_=*c*_*b*_ as discussed above and of course the custodial symmetry relation *c*_*V*_=*c*_*W*_=*c*_*Z*_ which holds in supersymmetric models. The results of this fit are presented in Fig. 4 for *c*_*t*_,*c*_*b*_,*c*_*V*_≥0, as motivated by the supersymmetric structure of the Higgs couplings (there is also an exact reflection symmetry under, *c*→−*c* or equivalently *β*→*β*+*π*, leaving the squared amplitudes of the Higgs rates unaffected). Again following Refs. [22–24], we have treated the theoretical uncertainty as a bias and not as if it were associated to a statistical distribution and have performed the fit for values of the signal strength $\mu_{i} \vert_{ \rm exp} [ 1 \pm\varDelta \mu _{i}/\mu_{i} \vert_{\rm th} ]$ with the theoretical uncertainty $\varDelta \mu_{i}/ \mu_{i} \vert_{\rm th}$ conservatively assumed to be 20 % for both the gluon and the vector boson fusion mechanisms (because of contamination) and ≈5 % for *h* production in association with *V*=*W*/*Z* [44, 45].

The best-fit value for the couplings, when the ATLAS and CMS data are combined, is *c*_*t*_=0.89,*c*_*b*_=1.01 and *c*_*V*_=1.02 with *χ* ^2^=64.8 (*χ* ^2^=66.7 in the SM).

**Fig. 5:**
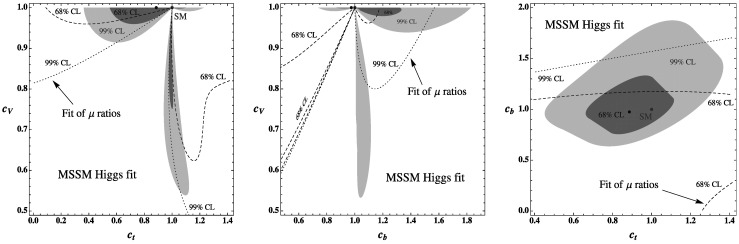
Best-fit regions at $68\ \%\ {\rm CL}$ (*green*) and $99\ \%\ {\rm CL}$ (*light gray*) for the Higgs signal strengths in the planes [*c*_*t*_,*c*_*V*_] (*left*), [*c*_*b*_,*c*_*V*_] (*center*) and [*c*_*t*_,*c*_*b*_] (*right*). The theoretical uncertainty on the Higgs signal strengths is taken into account as a bias. The best-fit contours at $68\ \%\ {\rm CL}$ (*dashed*) and $99\ \%\ {\rm CL}$ (*dotted*) from the fit of signal strength ratios are superimposed as well. The SM points are indicated *in red* and the best-fit points *in blue* (Color figure online)

In turn, in scenarios where the direct corrections in Eqs. (7)–(8) are not quantitatively significant (i.e. considering either not too large values of *μ*tan*β* or high stop/sbottom masses), one can use the MSSM relations of Eq. (6) to reduce the number of effective parameters down to two. For instance, using *c*_*t*_=cos*α*/sin*β* and *c*_*V*_=sin(*β*−*α*), one can derive the following relation: *c*_*b*_≡−sin*α*/cos*β*=(1−*c*_*V*_ *c*_*t*_)/(*c*_*V*_−*c*_*t*_). This allows to perform the two-parameter fit in the plane [*c*_*V*_,*c*_*t*_]. Similarly, one can study the planes [*c*_*V*_,*c*_*b*_] and [*c*_*t*_,*c*_*b*_]. The two-dimensional fits in these three planes are displayed in Fig. 5. As in the MSSM one has *α*∈[−*π*/2,0] and tan*β*∈[1,∼50], one obtains the following variation ranges: *c*_*V*_∈[0,1], $c_{t} \in[0,\sqrt{2}]$ and *c*_*b*_>0.

We also show on these figures the potential constraints obtained from fitting ratios of the Higgs signal strengths (essentially the two ratios *R*_*γγ*_=*μ*_*γγ*_/*μ*_*ZZ*_ and *R*_*ττ*_=*μ*_*ττ*_/*μ*_*WW*_) that are not or much less affected by the QCD uncertainties at the production level [22–24]. In this two-dimensional case, the best-fit points are located at (*c*_*t*_=0.88, *c*_*V*_=1.0), (*c*_*b*_=0.97, *c*_*V*_=1.0) and (*c*_*t*_=0.88, *c*_*b*_=0.97). Note that although for the best-fit point one has *c*_*b*_≲1, actually *c*_*b*_≳1 in most of the 1*σ* region.

**Fig. 6:**
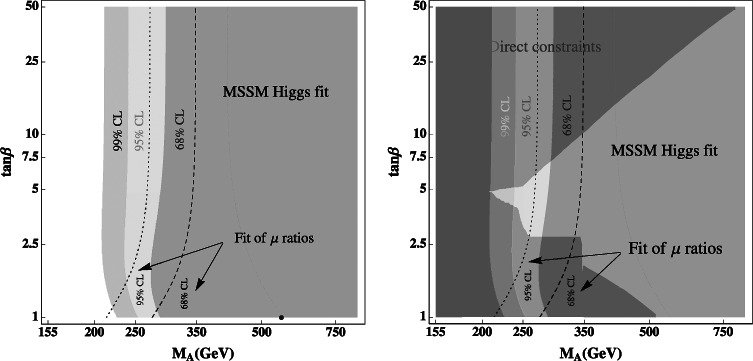
*Left*: best-fit regions at $68\ \%\ {\rm CL}$ (*green*), $95\ \%\ {\rm CL}$ (*yellow*) and $99\ \%\ {\rm CL}$ (*light gray*) for the Higgs signal strengths in the plane [tan*β*,*M*_*A*_]; the best-fit point is shown *in blue* and the theoretical uncertainty is taken into account as a bias as in the previous figures. The best-fit contours at 1*σ* (*dashed*) and 2*σ* (*dotted*) for the signal strength ratios are also shown. *Right*: we superimpose on these constraints the excluded regions (*in red*, and as a *shadow* when superimposed on the best-fit regions) from the direct searches of the heavier Higgs bosons at the LHC following the analysis of Ref. [15] (Color figure online)

Alternatively, using the expressions of Eq. (6), one can also realize a two-parameter fit in the [tan*β*,*α*] plane^6^ and with the expressions of Eq. (5) for the mixing angle *α* and fixing *M*_*h*_ to the measured value *M*_*h*_≈125 GeV, one can even perform a fit in the plane [tan*β*,*M*_*A*_]. This is shown in the left-hand side of Fig. 6 where the 68 % CL, 95 % CL and 99 % CL contours from the signal strengths only are displayed when, again, the theoretical uncertainty is considered as a bias. We also display the best-fit contours for the signal strength ratios at the 68 % CL and 95 % CL. The best-fit point for the signal strengths when the theoretical uncertainty is set to zero, is obtained for the values tan*β*=1 and *M*_*A*_=557 GeV. One should note, however, that the *χ* ^2^ value is relatively flat all over the 1*σ* region shown in Fig. 6. Hence, larger values of tan*β* and lower values of *M*_*A*_ could also be accommodated reasonably well by the fit. In any case, the best-fit point when taken literally, implies for the other parameters (using the information *M*_*h*_=125 GeV to derive the radiative corrections): *M*_*H*_=580 GeV, $M_{H^{\pm}}= 563$ GeV and $\alpha=-0.837~{\rm rad}$ which leads to cos(*β*−*α*)≃−0.05. Such a point with tan*β*≈1 implies an extremely large value of the SUSY scale, $M_{S} = \mathcal{ O}(100)$ TeV, for *M*_*h*_≈125 GeV.

It is interesting to superimpose on these indirect limits in the [tan*β*,*M*_*A*_] plane, the direct constraints on the heavy *H*/*A*/*H* ^±^ boson searches performed by the ATLAS and CMS collaborations as shown in the right-hand side of Fig. 6. As discussed in Ref. [15] (see also Ref. [46]), besides the limits from the *A*/*H*→*τ* ^+^ *τ* ^−^ and to a lesser extent *t*→*bH* ^+^→*bτν* searches which exclude high tan*β* values and which can be extended to very low tan*β* as well, there are also limits from adapting to the MSSM the high mass SM Higgs searches in the channels^7^ *H*→*WW* and *ZZ* as well as the searches for heavy resonances decaying into $t\bar{t}$ final states that exclude low values of tan*β* and *M*_*A*_. For values 250≲*M*_*A*_≲350 GeV, only the intermediate tan*β*≈2–10 range is still allowed.

## Conclusion

We have discussed the hMSSM, i.e. the MSSM that we seem to have after the discovery of the Higgs boson at the LHC that we identify with the lighter *h* state. The mass *M*_*h*_≈125 GeV and the non-observation of SUSY particles, seems to indicate that the soft-SUSY breaking scale might be large, *M*_*S*_≳1 TeV. We have shown, using both approximate analytical formulas and a scan of the MSSM parameters, that the MSSM Higgs sector can be described to a good approximation by only the two parameters tan*β* and *M*_*A*_ if the information *M*_*h*_=125 GeV is used. One could then ignore the radiative corrections to the Higgs masses and their complicated dependence on the MSSM parameters and use a simple formula to derive the other parameters of the Higgs sector, *α*, *M*_*H*_ and $M_{H^{\pm}}$. This will considerably simplify phenomenological analyses in the MSSM which up to now rely either on large scans of the parameter space (as e.g. Refs. [26, 27]) or resort to benchmark scenarios in which most of the MSSM parameters are fixed (as is the case of Ref. [28] for instance).

In a second step, we have shown that to describe accurately the *h* properties when the direct radiative corrections are also important, the three couplings *c*_*t*_,*c*_*b*_ and *c*_*V*_ are needed besides the *h* mass. We have performed a fit of these couplings using the latest LHC data and taking into account properly the theoretical uncertainties. The fit turns out to very slightly favor the low tan*β* region with a not too high CP-odd Higgs mass.

**Fig. 7:**
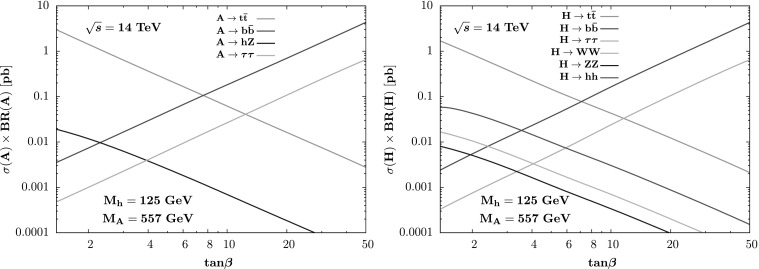
The cross section times branching fractions for the *A* (*left*) and *H* (*right*) MSSM Higgs bosons at the LHC with $\sqrt{s}=14$ TeV as a function of tan*β* for the best-fit mass *M*_*A*_=557 GeV and with *M*_*h*_=125 GeV. For the production, we have taken into account only the gluon and bottom quark fusion processes and followed the analysis given in Ref. [15]

The phenomenology of this low tan*β* MSSM region is quite interesting. First, the heavier Higgs particles could be searched for in the next LHC run in the channels $A \to t \bar{t}, hZ$ and *ττ* and in the modes *H*→*WW*,*ZZ*,*hh* for which the rates can be substantial for $\tan\beta =\mathcal{ O}(1)$. This is shown in Fig. 7 where the cross sections times decay branching ratios for *A* and *H* are displayed as a function of tan*β* for the choice *M*_*A*_=557 GeV for $\sqrt{s}=14$ TeV. Furthermore, the correct relic abundance of the LSP neutralino can be easily obtained through $\chi_{1}^{0} \chi_{1}^{0} \to A \to t\bar{t}$ annihilation by allowing the parameters *μ* and *M*_1_ to be comparable and have an LSP mass close to the *A*-pole, $m_{\chi_{1}^{0}} \approx\frac{1}{2} M_{A}$. This low tan*β* region will be discussed in more detail in a separate publication [47].

## Appendix: Approximating the radiative corrections

The radiative corrections to the CP-even Higgs boson mass matrix can be written as

A.1 $$\begin{aligned} \mathcal{ M}^2 = \left [ \begin{array}{c@{\quad }c} \mathcal{ M}_{11}^2 + \varDelta \mathcal{ M}_{11}^2 & \mathcal{ M}_{12}^2 + \varDelta \mathcal{ M}_{12}^2 \\ \mathcal{ M}_{12}^2 + \varDelta \mathcal{ M}_{12}^2 & \mathcal{ M}_{22}^2 + \varDelta \mathcal{ M}_{22}^2 \end{array} \right ] \end{aligned}$$

The leading one-loop radiative corrections $\varDelta \mathcal{ M}_{ij}^{2}$ to the mass matrix are controlled by the top Yukawa coupling *λ*_*t*_=*m*_*t*_/*v*sin*β* which appears with the fourth power. One can obtain a very simple analytical expression if only this contribution is taken into account [7–9]:

A.2 $$ \begin{aligned} & \varDelta \mathcal{ M}_{11}^2 \sim \varDelta \mathcal{ M}_{12}^2 \sim0 \\ &\varDelta \mathcal{ M}_{22}^2 \sim \epsilon\\ &\hphantom{\varDelta \mathcal{ M}_{22}^2} = \frac{3 \bar {m}_t^4}{2\pi^2 v^2\sin^ 2\beta} \biggl[ \log\frac{M_S^2}{\bar{m}_t^2} + \frac{X_t^2}{M_S^2} \biggl( 1 - \frac{X_t^2}{12M_S^2} \biggr) \biggr]\end{aligned} $$

where *M*_*S*_ is the geometric average of the stop masses $M_{S} =\sqrt{ m_{\tilde{t}_{1}}m_{\tilde{t}_{2}}} $, *X*_*t*_ is the stop mixing parameter given by *X*_*t*_=*A*_*t*_−*μ*/tan*β* and $\bar{m}_{t}$ is the running ${\rm \overline{MS}}$ top quark mass to account for the leading two-loop QCD corrections in a renormalization-group improvement.

A better approximation, with some more renormalization-group improved two-loop QCD and electroweak corrections included is given by [10, 11]

A.3 $$\begin{aligned} \varDelta \mathcal{ M}_{22}^2 =& \frac{3}{2\pi^2} \frac{m_t^4}{v^2 \sin ^2\beta} \biggl[ \frac{1}{2} \tilde{X}_t + \ell_S \\ &{} +\frac{1}{16\pi^2} \biggl(\frac{3}{2} \frac{m_t^2}{v^2}-32\pi\alpha_s \biggr) \bigl(\tilde{X}_t \ell_S+ \ell_S^2 \bigr) \biggr] \end{aligned}$$

where $\ell_{S}= \log(M_{S}^{2}/m_{t}^{2})$ and using *x*_*t*_=*X*_*t*_/*M*_*S*_=(*A*_*t*_−*μ*cot*β*)/*M*_*S*_ one has $\tilde{X}_{t} = 2 x_{t}^{2} (1 - x_{t}^{2}/12)$ with *A*_*t*_ the trilinear Higgs-stop coupling and *μ* the higgsino mass parameter.

Other soft SUSY-breaking parameters, in particular *μ* and *A*_*b*_ (and in general the corrections controlled by the bottom Yukawa coupling *λ*_*b*_=*m*_*b*_/*v*cos*β* which at large value of the product *μ*tan*β*, provide a non-negligible correction to $\mathcal{ M}_{ij}^{2}$) can also have an impact on the loop corrections. Including these subleading contributions at one-loop, plus the leading logarithmic contributions at two loops, the radiative corrections to the CP-even mass matrix elements can still be written in a compact form [6]

A.4 $$ \begin{aligned} \varDelta \mathcal{ M}_{11}^2 &= - \frac{v^2 \sin^2\beta}{32 \pi^2} \bar { \mu}^2 \bigl[ x_t^2 \lambda_t^4 (1+ c_{11} \ell_S)\\ &\quad {} + a_b^2 \lambda_b^4 (1+ c_{12} \ell_S) \bigr] \\ \varDelta \mathcal{ M}_{12}^2 &= - \frac{v^2 \sin^2\beta}{32 \pi^2} \bar {\mu} \bigl[ x_t \lambda_t^4 (6- x_t a_t) (1+ c_{31} \ell_S)\\ &\quad {}- \bar{\mu}^2 a_b \lambda_b^4 (1+ c_{32} \ell_S) \bigr] \\ \varDelta \mathcal{ M}_{22}^2 &= \frac{v^2 \sin^2\beta}{32 \pi^2} \bigl[ 6 \lambda_t^4 \ell_S (2+ c_{21} \ell_S)\\ &\quad {} + x_t a_t \lambda_t^4 (12 - x_t a_t) (1+ c_{21} \ell_S)\\ &\quad {} - \bar{\mu}^4 \lambda_b^4 (1+ c_{22} \ell_S) \bigr] \end{aligned}$$

where the additional abbreviations $\bar{\mu}=\mu/M_{S}$ and *a*_*t*,*b*_=*A*_*t*,*b*_/*M*_*S*_ have been used. The factors *c*_*ij*_ take into account the leading two-loop corrections due to the top and bottom Yukawa couplings and to the strong coupling constant $g_{s}=\sqrt{4\pi \alpha_{s}}$; they read

A.5 $$\begin{aligned} c_{ij}= \frac{1}{32\pi^2} \bigl(t_{ij}\lambda_{t}^2+ b_{ij}\lambda_{b}^2 -32 g_s^2 \bigr) \end{aligned}$$

with the various coefficients given by (*t*_11_,*t*_12_,*t*_21_,*t*_22_,*t*_31_,*t*_32_)=(12,−4,6,−10,9,7) and (*b*_11_,*b*_12_,*b*_21_,*b*_22_,*b*_31_,*b*_32_)=(−4,12,2,18,−1,15).

The expressions Eq. (A.4) provide a good approximation of the bulk of the radiative corrections [6]. However, one needs to include the full set of corrections to have precise predictions for the Higgs boson masses and couplings as discussed at the end of Sect. 2.

## Footnotes

1 We will assume here that all the MSSM parameters are real. The CP-violating MSSM needs a separate discussion which is beyond the scope of the present paper.

2 A different definition for the mixing angle *α*, namely $\alpha\to\frac{\pi}{2}- \alpha$, has been adopted in Refs. [13, 14, 21].

3 The physics of the charged boson, i.e. the production and decay rates, can be accurately described by $\tan\beta, M_{H^{\pm}}$ (and eventually *α* if the subleading processes involving the *h* state are also considered).

4 The chargino contribution cannot exceed the 10 % level even for very favorable gaugino–higgsino parameters [19], while the $\tilde{\tau}$ contributions are important only for extreme values of tan*β* and *μ* [32, 33].

5 Higgs coupling fits have been performed most often in the [*c*_*V*_,*c*_*f*_] parameter space with *c*_*f*_=*c*_*t*_=*c*_*b*_⋯ . Fits of the LHC data in SUSY scenarios including also the NMSSM can be found in Refs. [36–43] for instance.

6 This corresponds in fact to the case of a two-Higgs doublet model in which the direct corrections are expected to be small in contrast to the SUSY case: one can then parametrize the couplings of the *h* boson, which are given by Eq. (6), by still two parameters *α* and *β* but with the angle *α* being a free input.

7 At low tan*β*, channels such as *A*→*hZ* and *H*→*hh* need also to be considered [15]. In the latter case, special care is needed in the treatment of the trilinear *Hhh* coupling as will be discussed in Ref. [47].
